# Supplementary material for: Structural basis for the interaction between the bacterial cell division proteins FtsZ and ZapA
Source: Nat Commun. 2025 Jul 1;16:5985. doi: 10.1038/s41467-025-60940-w (PMC12216130; doi:10.1038/s41467-025-60940-w)
Supplement: Supplementary file 1 — Supplementary Information [file 41467_2025_60940_MOESM1_ESM.pdf]

Supplementary Information for

Structural basis for the interaction between the bacterial cell division proteins FtsZ and ZapA

Junso Fujita[1,2,3,#], Kazuki Kasai[1,2,#], Kota Hibino[4], Gota Kagoshima[5], Natsuki Kamimura[4], Shungo Tobita[4], Yuki Kato[4], Ryo Uehara[4], Keiichi Namba[1,2], Takayuki Uchihashi[5,6,7,*], Hiroyoshi Matsumura[4,*]

[1]Graduate School of Frontier Biosciences, The University of Osaka, 1-3 Yamadaoka, Suita, Osaka 565-0871, Japan.

[2]JEOL YOKOGUSHI Research Alliance Laboratories, The University of Osaka, 1-3 Yamadaoka, Suita, Osaka 565-0871, Japan.

[3]Graduate School of Pharmaceutical Sciences, The University of Osaka, 1-6 Yamadaoka, Suita, Osaka 565-0871, Japan.

[4]Department of Biotechnology, College of Life Sciences, Ritsumeikan University, 1-1-1 Noji-higashi, Kusatsu, Shiga, 525-8577, Japan

[5]Department of Physics, Nagoya University, Furo-cho, Chikusa-ku, Nagoya, Aichi 464-8602, Japan

[6]Exploratory Research Center on Life and Living Systems (ExCELLS), National Institutes of Natural Sciences, Okazaki, Aichi 444-8787, Japan

[7]Institute for Glyco-core Research (iGCORE), Nagoya University, Nagoya, Aichi 464-0814, Japan

[#]These authors contributed equally: Junso Fujita, Kazuki Kasai

*Correspondence to: Takayuki Uchihashi (uchihast@d.phys.nagoya-u.ac.jp) and Hiroyoshi Matsumura (h-matsu@fc.ritsumei.ac.jp)

This supplementary information contains:

Supplementary Fig. 1–13

Supplementary Table 1–5

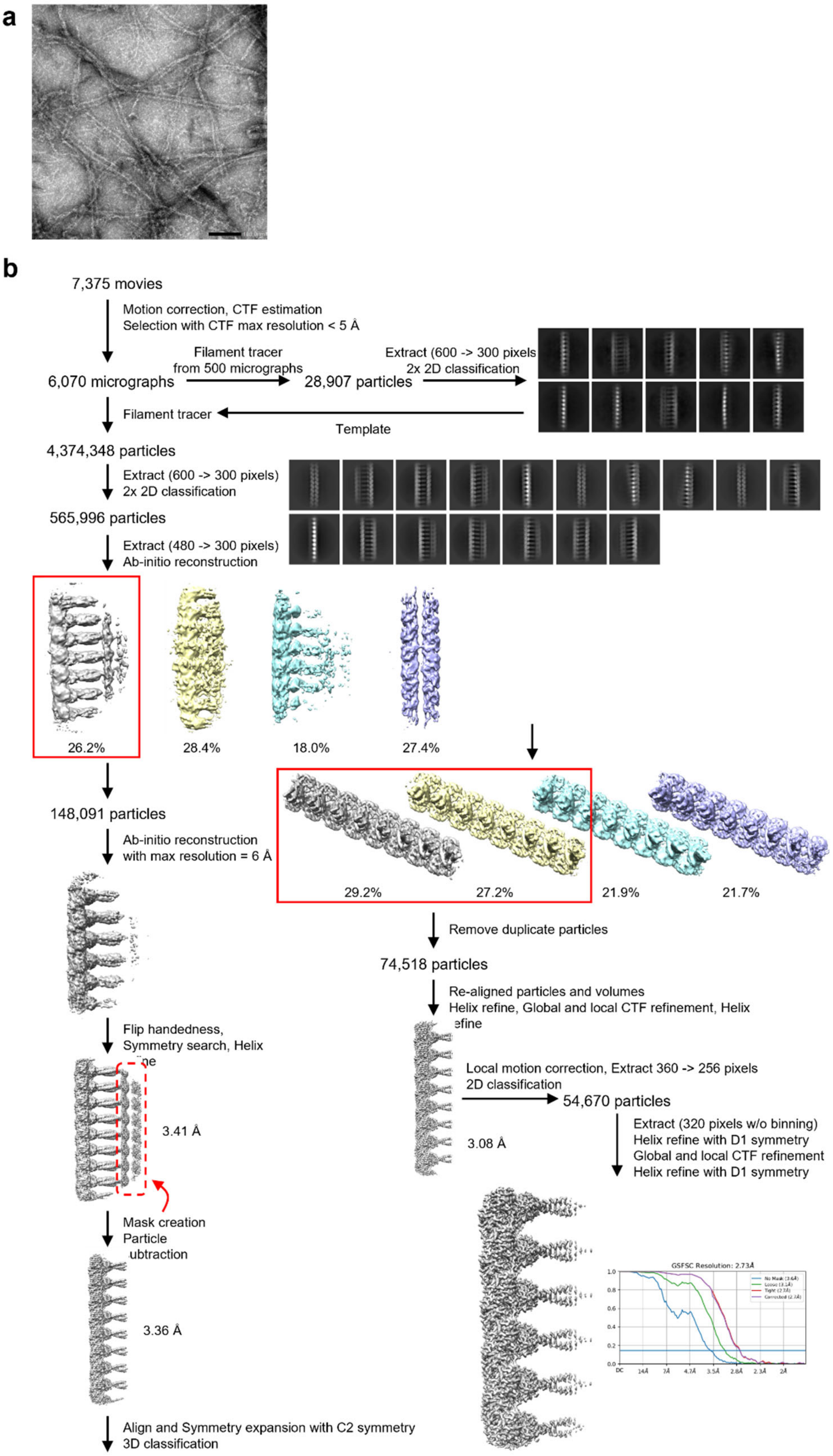

Supplementary Fig. 1: Representative negative stain EM image and cryo-EM data processing workflow of the KpFtsZ-ZapA complex. a, A negative stain EM image. The scale bar represents 100 nm. **b,** Cryo-EM data processing workflow of the KpFtsZ-ZapA complex.

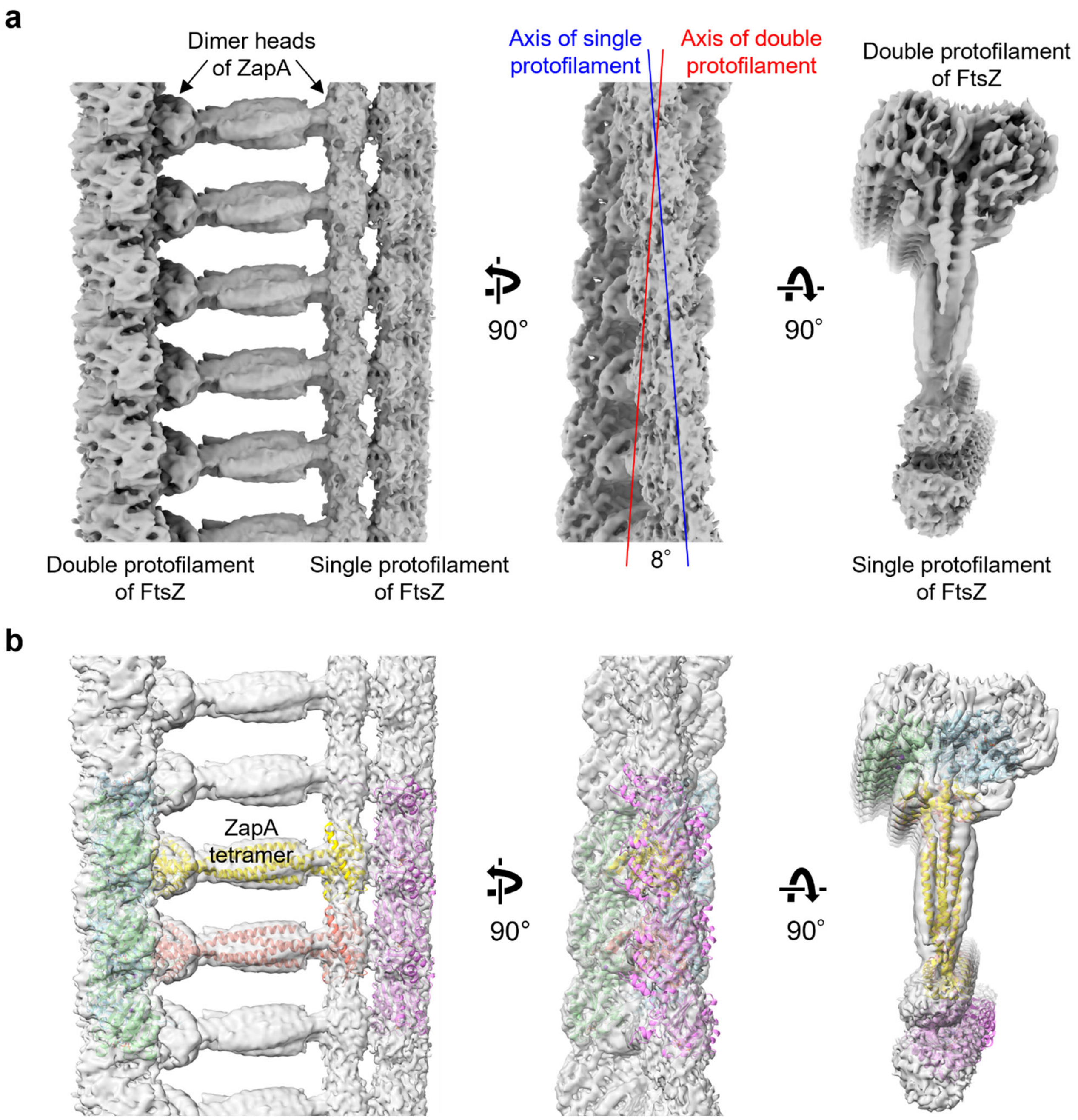

Supplementary Fig. 2: The cryo-EM map of KpFtsZ-ZapA complex. a, Three orthogonal views of the map. Axes of FtsZ double and single protofilaments are also shown in the middle panel. **b,** Fitted FtsZ and ZapA models with the same map in a. Each of the FtsZ protofilaments and ZapA tetramers is shown in different colors. Since the density map on the single protofilament side is rather blurred, the single protofilament and a ZapA dimer on this side are roughly fitted in this analysis.

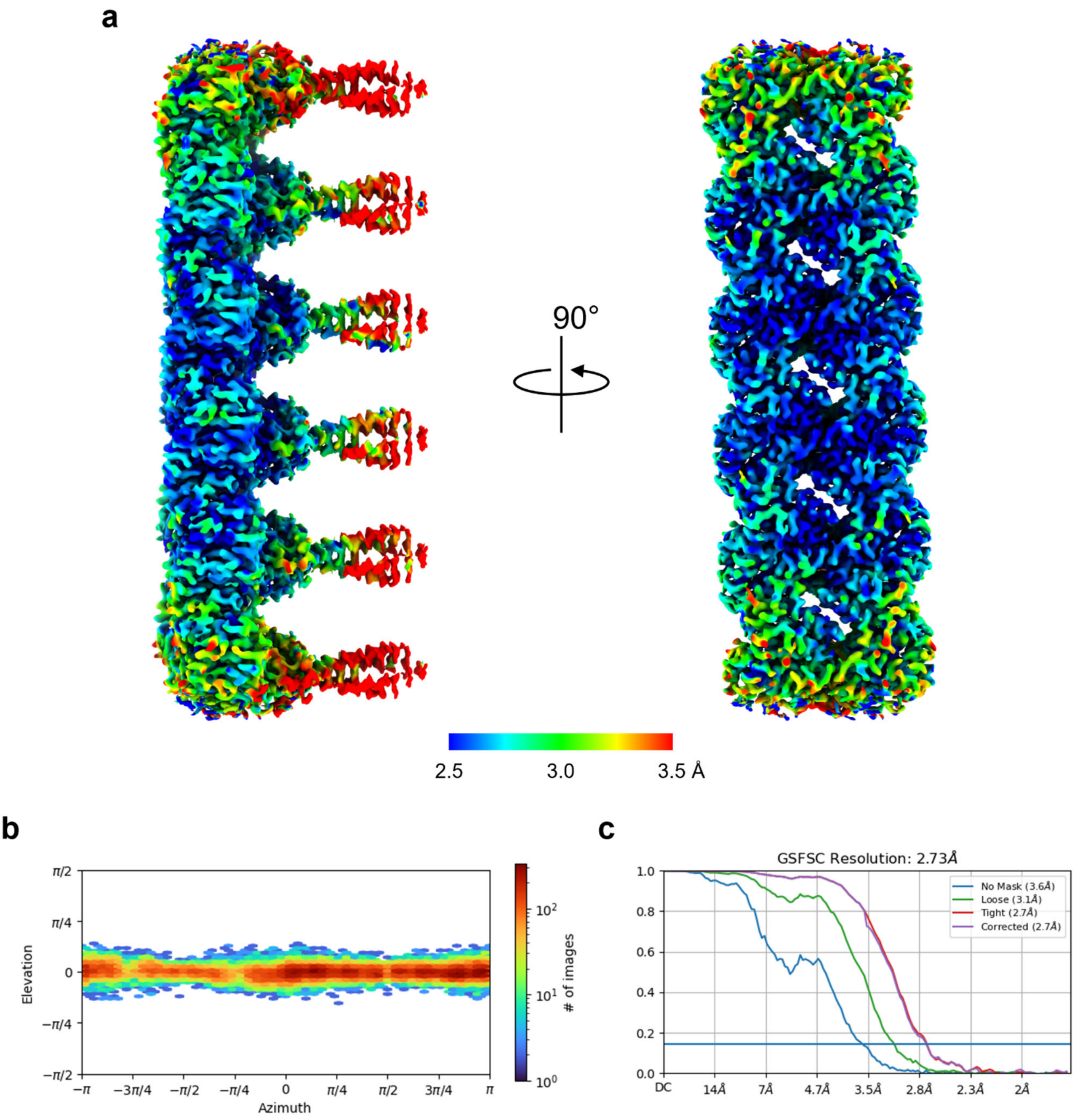

Supplementary Fig. 3: Cryo-EM data processing of the ZapA-FtsZ complex. a, Final sharpened map colored by local resolution as in the color bar. **b**, Angular distribution of the particles used in the final reconstruction. **c**, The FSC curve for the final map. The horizontal blue line indicates the FSC = 0.143 criterion.

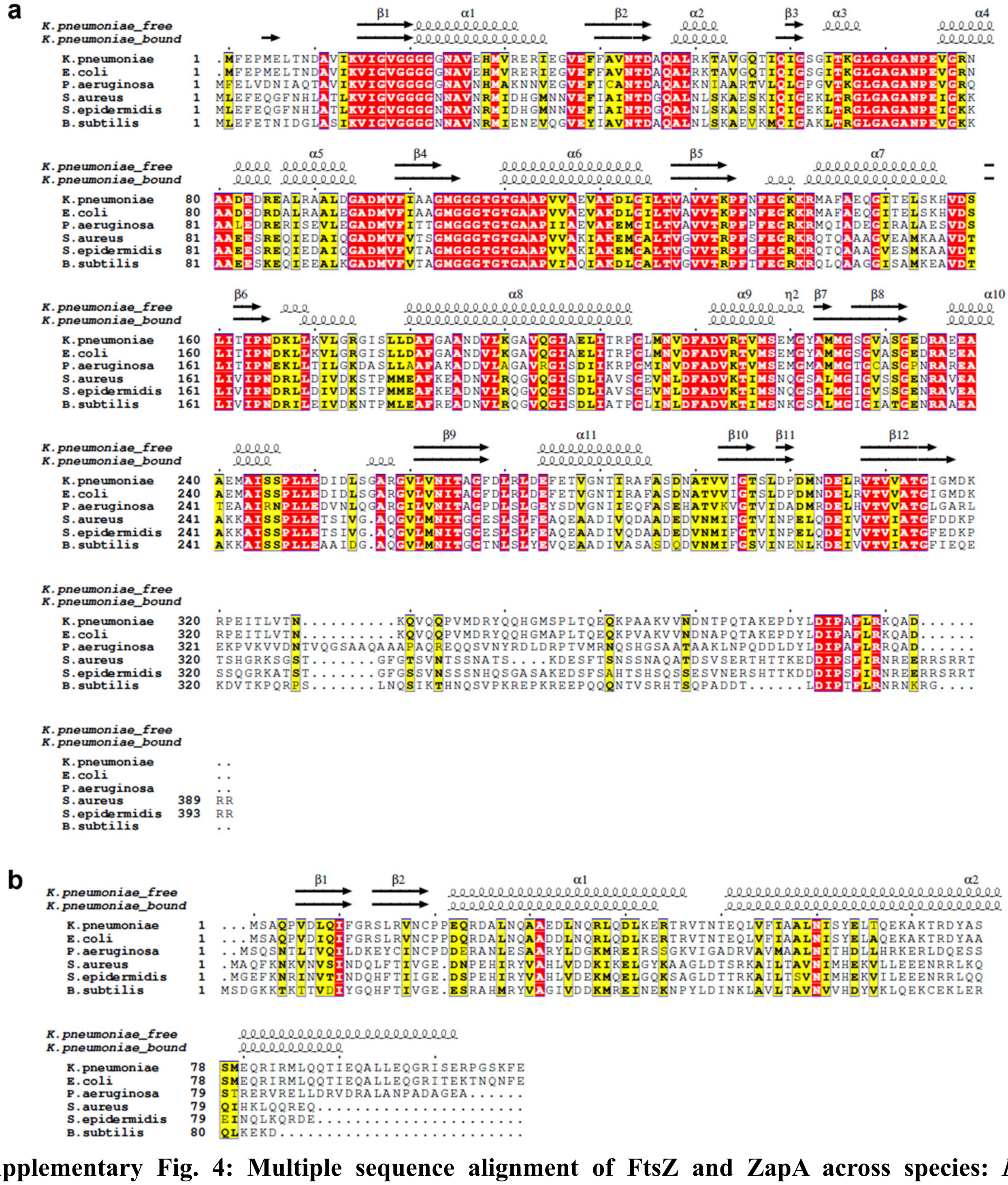

Supplementary Fig. 4: Multiple sequence alignment of FtsZ and ZapA across species: *K. pneumoniae* FtsZ (KpFtsZ; UniProt ID A6T4N8 [https://www.uniprot.org/uniprotkb/A6T4N8/entry]), *E. coli* FtsZ (EcFtsZ; UniProt ID P0A9A6 [https://www.uniprot.org/uniprotkb/P0A9A6/entry]), *P. aeruginosa* FtsZ (PaFtsZ; UniProt ID P47204 [https://www.uniprot.org/uniprotkb/P47204/entry]), *B. subtilis* FtsZ (BsFtsZ; UniProt ID P17865 [https://www.uniprot.org/uniprotkb/P17865/entry]), *S. aureus* FtsZ (SaFtsZ; UniProt ID Q6GHP9 [https://www.uniprot.org/uniprotkb/Q6GHP9/entry]) *S. epidermidis* FtsZ (SeFtsZ; UniProt ID Q8CPK4 [https://www.uniprot.org/uniprotkb/Q8CPK4/entry]), *K. pneumoniae* ZapA (KpZapA; UniProt ID B5XUC8

[https://www.uniprot.org/uniprotkb/B5XUC8/entry]), *E. coli* ZapA (EcZapA; UniProt ID P0ADS2 [https://www.uniprot.org/uniprotkb/P0ADS2/entry]), *P. aeruginosa* ZapA (PaZapA; UniProt ID Q9HTW3 [https://www.uniprot.org/uniprotkb/Q9HTW3/entry]), *B. subtilis* ZapA (BsZapA; UniProt ID W8URK0 [https://www.uniprot.org/uniprotkb/W8URK0/entry]), *S. aureus* ZapA (SaZapA; UniProt ID A0A380DNW5 [https://www.uniprot.org/uniprotkb/A0A380DNW5/entry]) and *S. epidermidis* ZapA (SeZapA; UniProt ID A0A0N1EE61 [https://www.uniprot.org/uniprotkb/A0A0N1EE61/entry]).

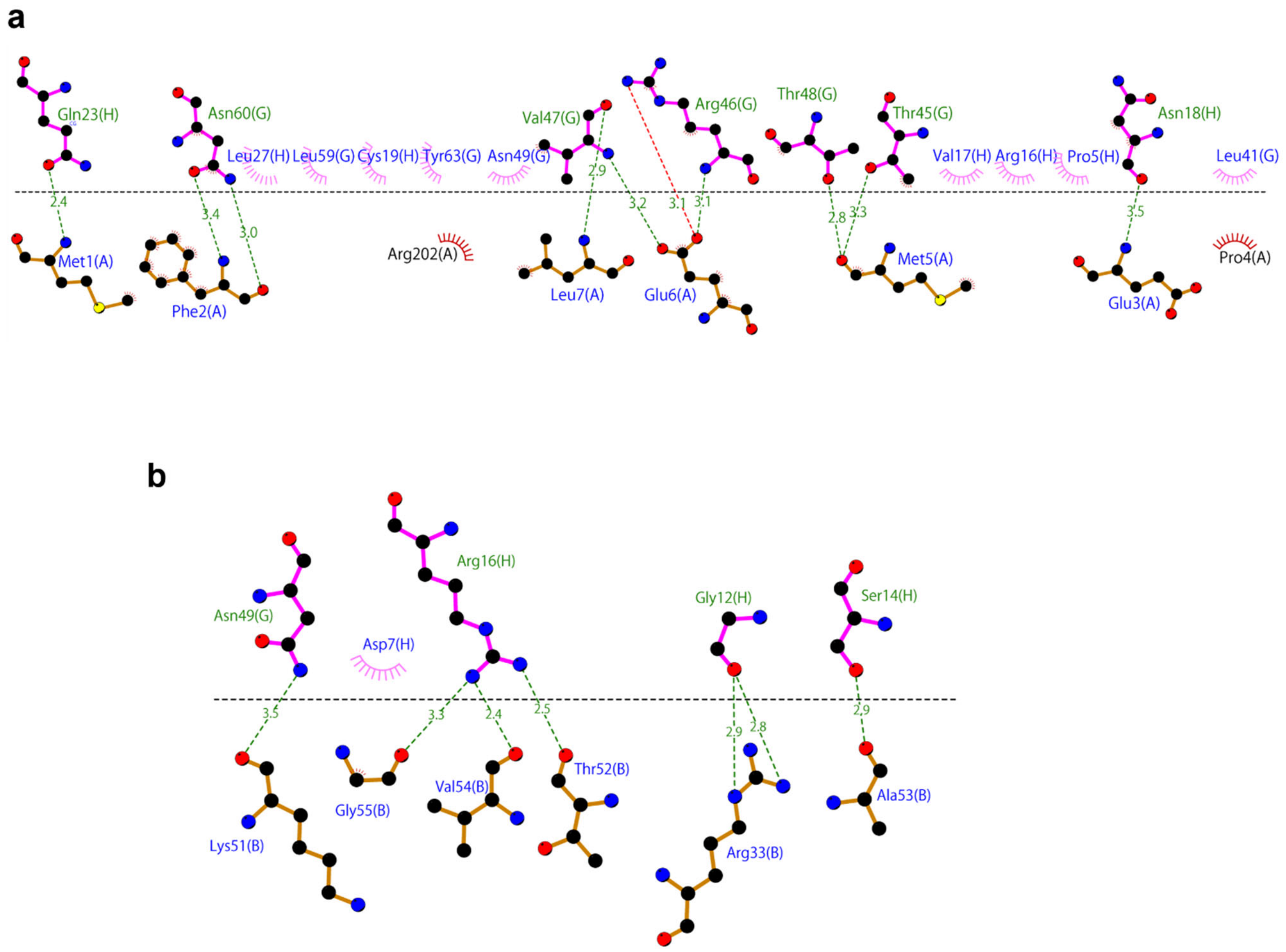

Supplementary Fig. 5: Detailed description of the interactions generated with the program Ligplot. a, Interactions between ZapA (chains G and H) and FtsZ (chain A). Hydrogen bonds are represented by dashed lines. **b**, Interactions between ZapA (chains G and H) and FtsZ (chain B).

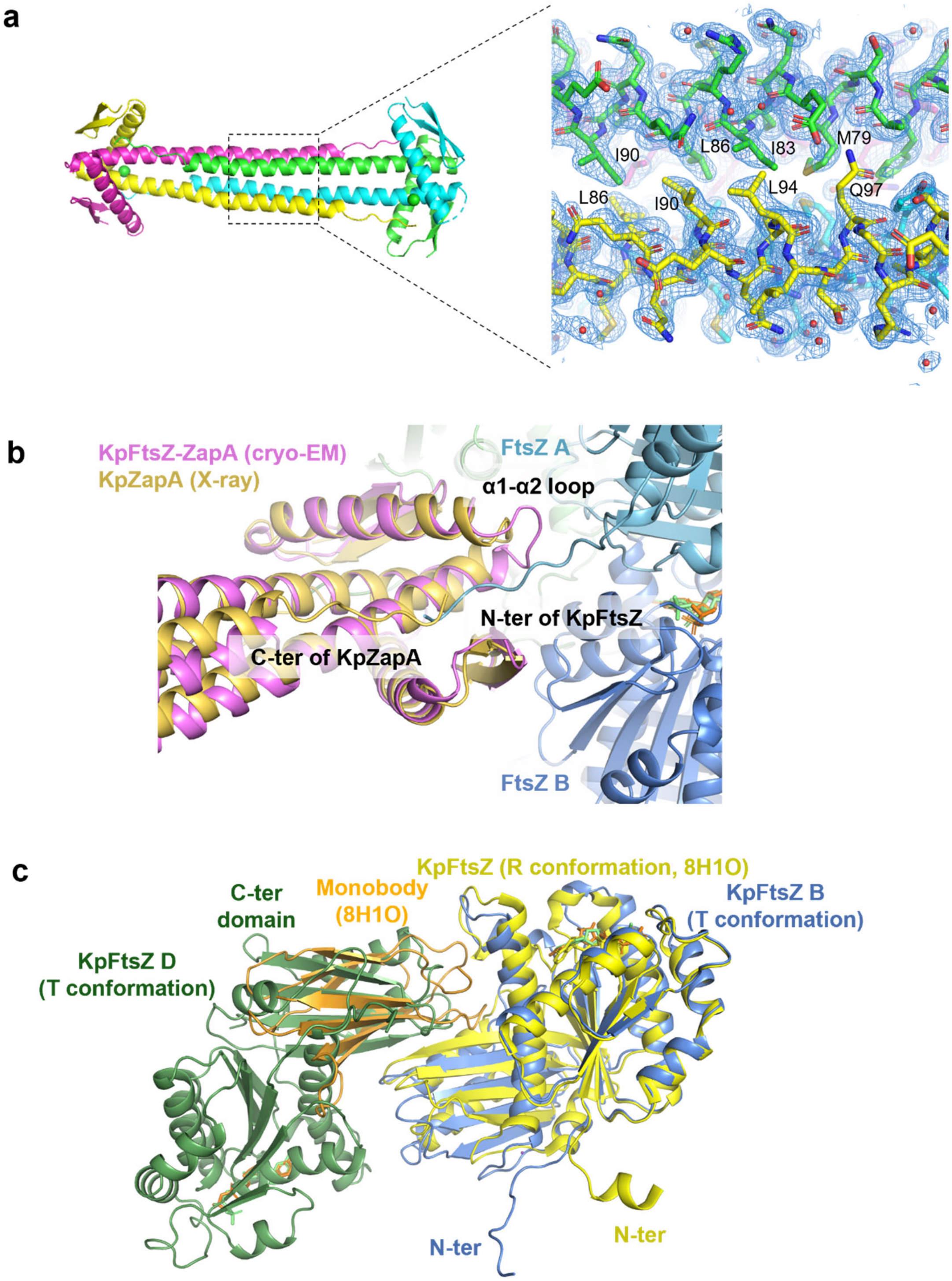

Supplementary Fig. 6: Structural comparison of FtsZ within the ZapA-FtsZ complex. a, The crystal structure of the KpFtsZ tetramer at 1.8 Å resolution. The 2Fo-Fc electron-density (blue) around the coiled-coil region is contoured at 1σ. **b,** Overlapping of N-terminus of KpFtsZ and C-terminus of KpZapA. The coloring is the same as in Fig. 2c. **c,** Overlapping of C-terminal domain in KpFtsZ from another protofilament and the monobody bound to KpFtsZ in the R conformation (PDB id: 8H1O [https://doi.org/10.2210/pdb8H1O/pdb]).

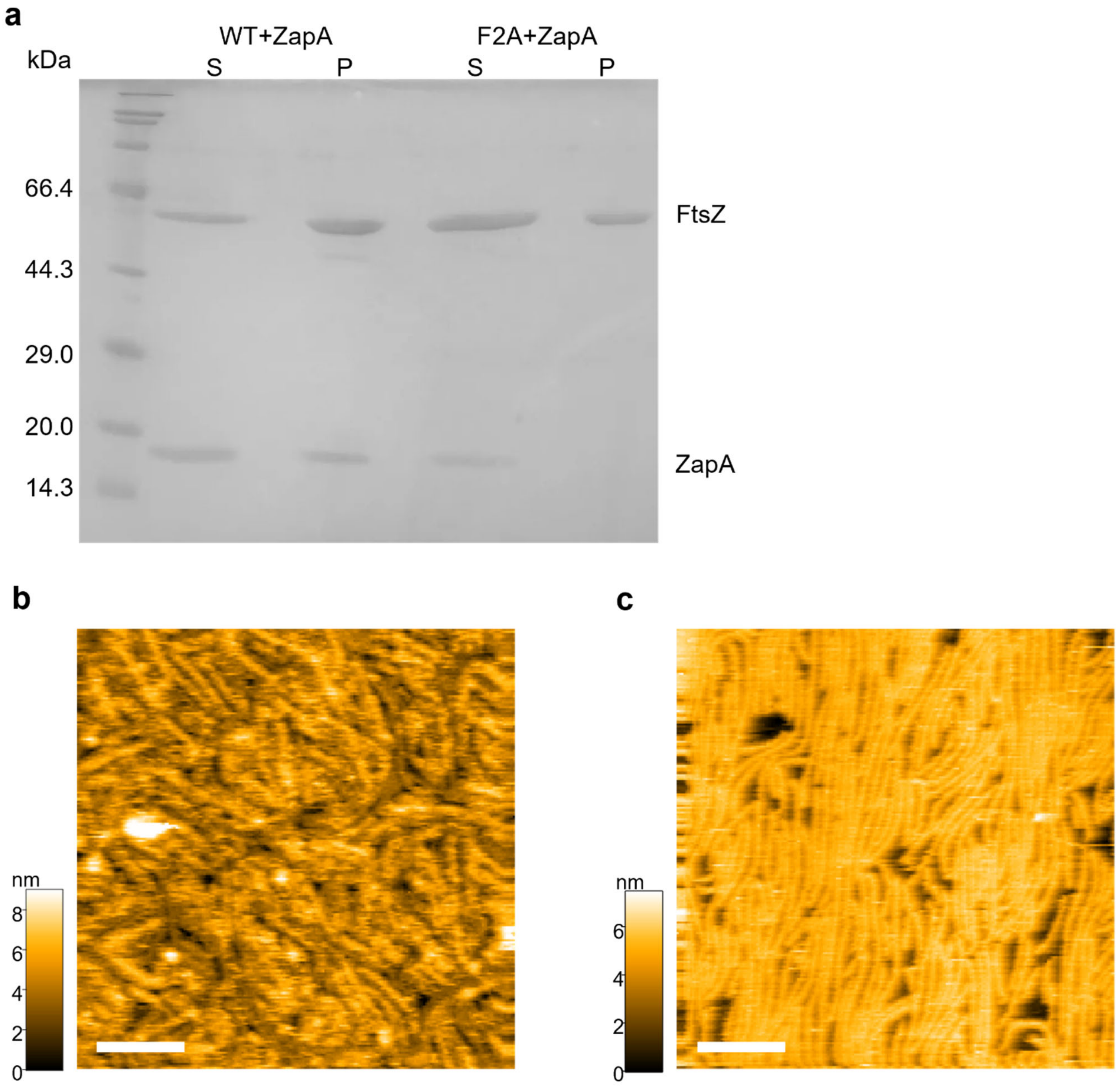

Supplementary Fig. 7: Interaction analysis using sedimentation assays and HS-AFM. a, Coomassie-stained SDS-PAGE gel. Polymerization of FtsZ (upper) and ZapA (lower) at 86 mM KCl. (S) supernatant, (P) pellet fractions from the experiment. **b, c**, Interaction between wild-type and F2A mutant with ZapA. HS-AFM images of mixed samples (ZapA addition method) of **b**, FtsZ-WT (0.1 mM GMPCPP, 13 µM KpZapA) and **c** FtsZ-F2A (1 mM GMPCPP, 27 µM KpZapA) with ZapA. Scale bar: 100 nm, frame rate: 1 frame per second (fps), 200 × 200 pixels. The results shown in the two HS-AFM images were obtained from more than 3 independent experiments.

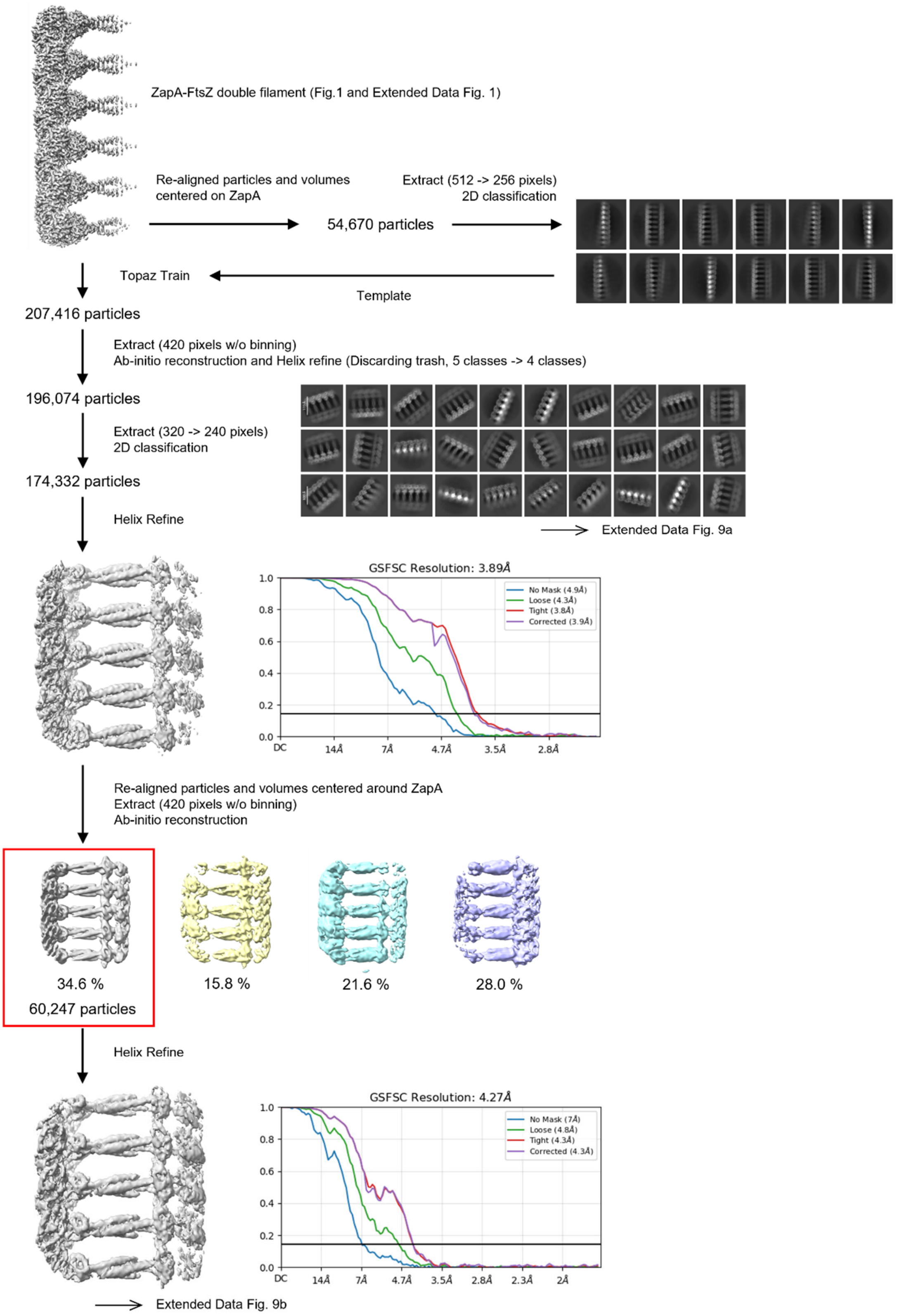

Supplementary Fig. 8: Cryo-EM data processing workflow of KpFtsZ-ZapA-FtsZ complex focused on ZapA. Cryo-EM data re-processing workflow of the KpFtsZ-ZapA complex focused on ZapA. The 3D reconstructions at 3.89 Å resolution and 4.27 Å are nearly identical, and the FtsZ single protofilament region is more clearly defined at 4.27 Å.

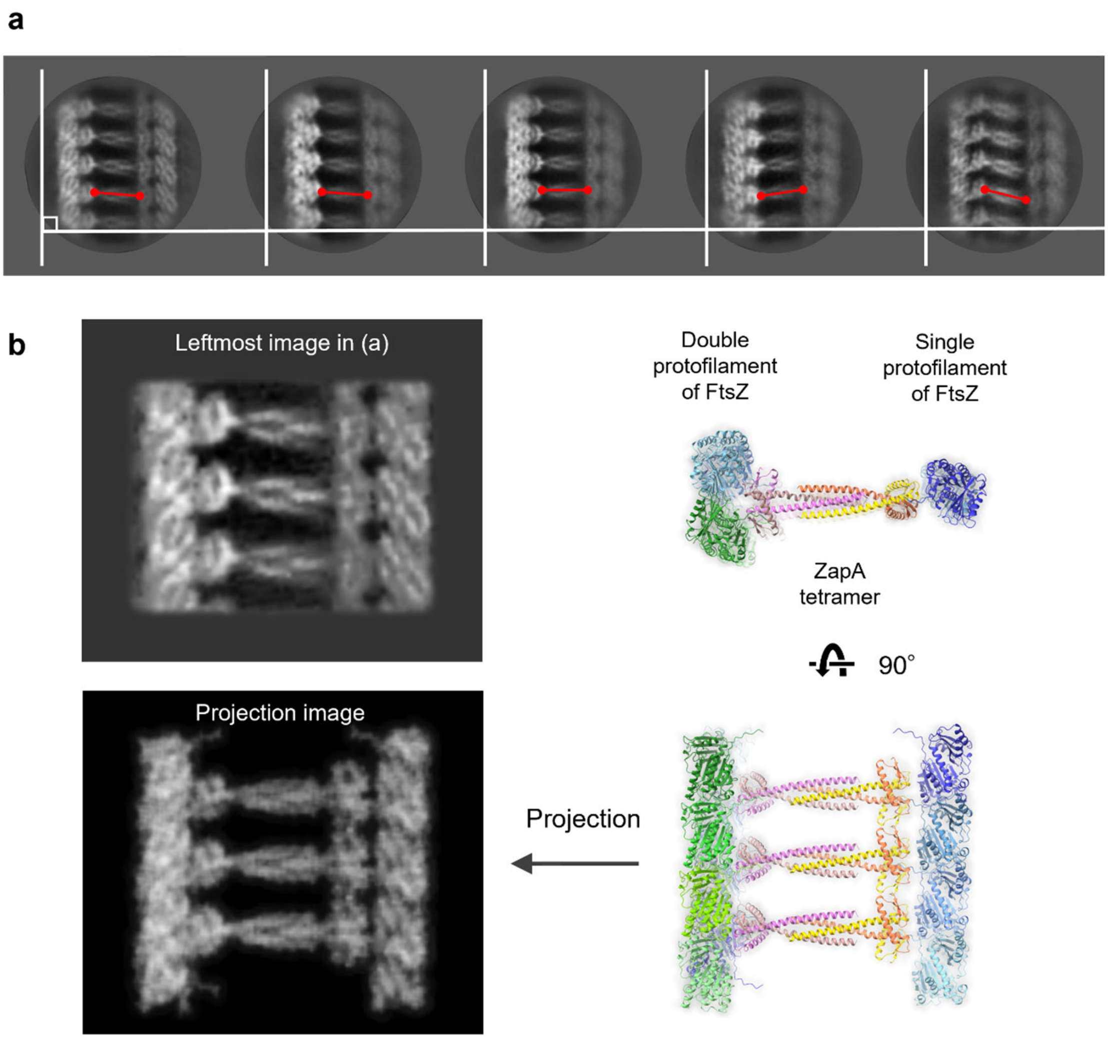

Supplementary Fig. 9: Re-analysis of the images of FtsZ-ZapA-FtsZ complex focused on ZapA. **a,** Five classes of 2D average images of the FtsZ-ZapA-FtsZ complex centered on ZapA. These images were also used to reconstruct the 3D structure and determine the symmetry of ZapA tetramer. The white lines indicate the vertical and horizontal axes. The red lines indicate the orientation of ZapA tetramer, with red circles at both ends indicating the positions of junctions of the C-terminal head and coiled-coil domain of ZapA dimer. **b,** A model of FtsZ-ZapA-FtsZ complex predicted from the 2D class average images shown in (**a**). Top left: an enlarged view of the leftmost image of the five in (**a**). Top right and bottom right: a ribbon model of the complex formed by FtsZ double protofilaments (left), ZapA tetramer (middle), and FtsZ single protofilament (right), including the one determined by 3D reconstruction at 2.73 Å resolution for the left half of the complex. The model was built by first fitting the model of ZapA dimer and FtsZ double protofilament to the EM map of ZapA tetramer reconstructed separately. Then, an FtsZ single protofilament taken from the double protofilament was placed to reproduce the secondary structure pattern of FtsZ in the single protofilament image of the top left. Bottom left: a projection map generated from the model on the right for comparison with the 2D average image at the top left.

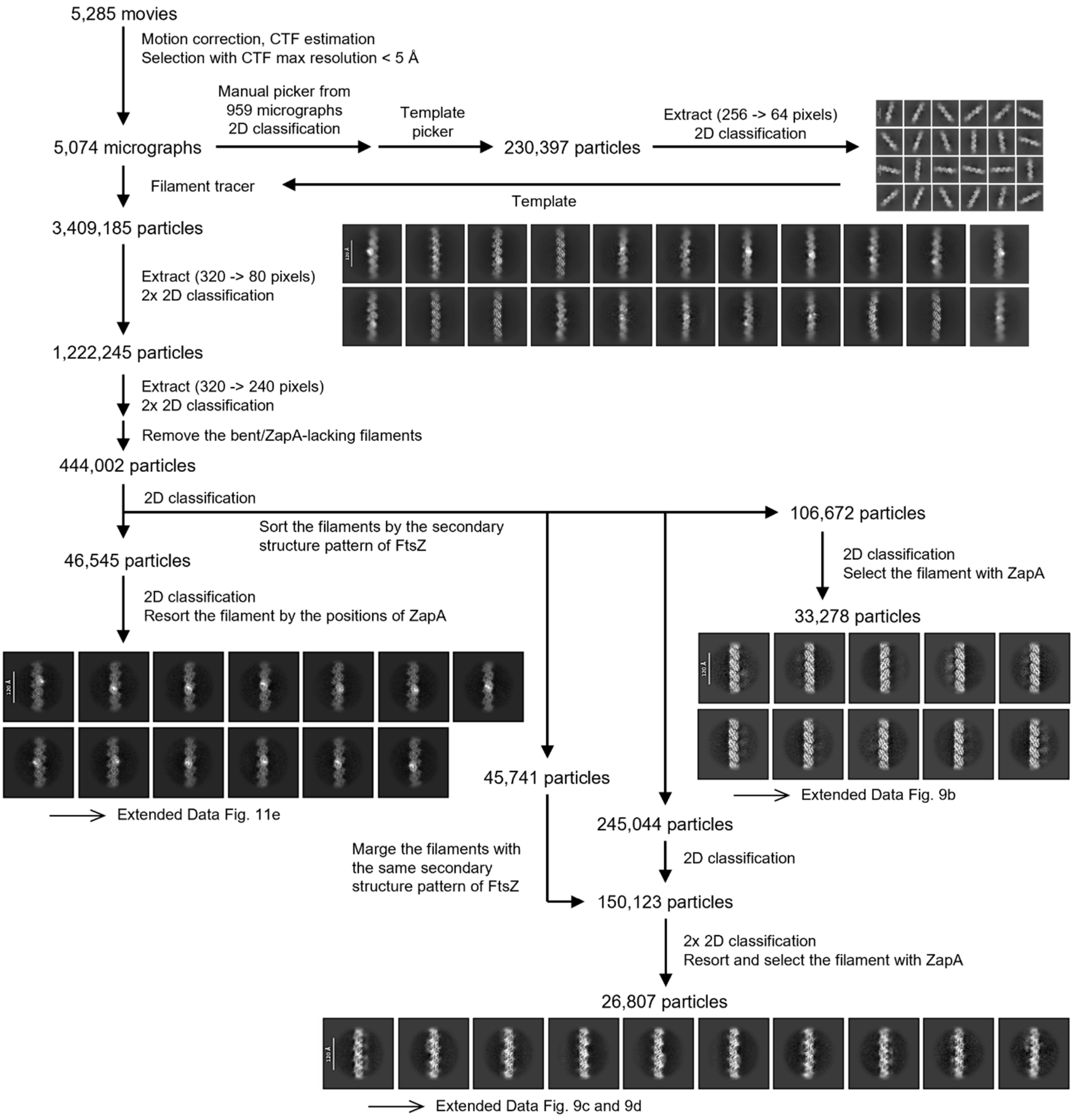

Supplementary Fig. 10: Cryo-EM data processing workflow of the FtsZ single protofilament with a mutant ZapA (E83I).

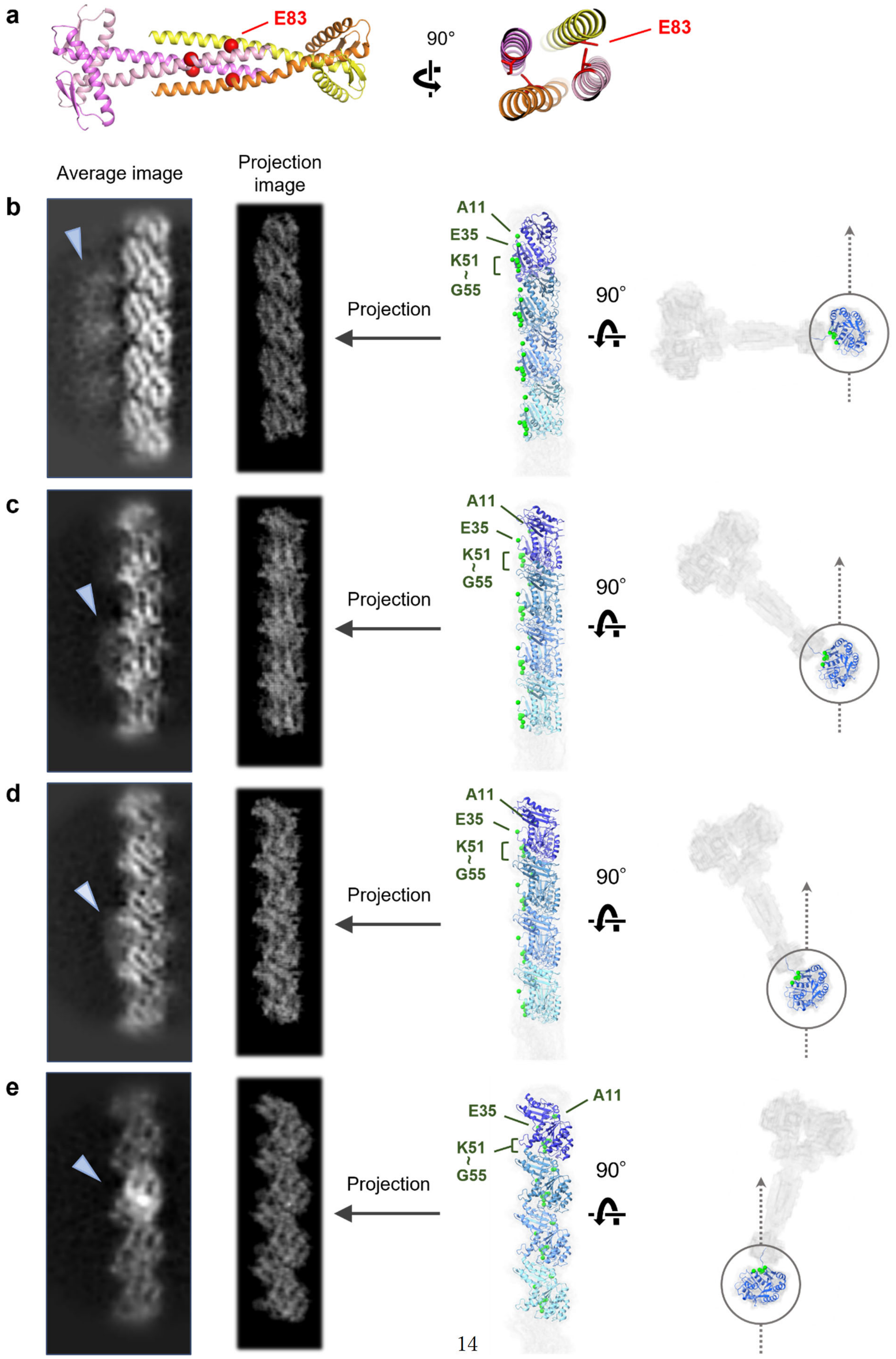

a
E83
90°
E83
Average image
Projection image
b
A11
E35
K51
~
G55
Projection
90°
c
A11
E35
K51
~
G55
Projection
90°
d
A11
E35
K51
~
G55
Projection
90°
e
A11
E35
K51
~
G55
Projection
90°

Supplementary Fig. 11: 2D class images of the FtsZ single protofilament with a mutant ZapA (E83I) dimer showing their weak interactions. a, A ribbon model of ZapA tetramer with the E83 residue highlighted as red spheres or sticks. The E83I replacement inhibits the interaction between ZapA dimers in the coiled-coil region to form the tetramer; therefore, ZapA(E83I) exists as a dimer with a single dimer head. **b-e,** 2D class average images of the ZapA(E83I)-FtsZ single protofilament complex on the left, ribbon models of FtsZ single protofilament on the right in the azimuthal orientations that reproduce the secondary structure patterns of FtsZ in the average images, and projection maps of these models on the second left for comparison with the average images on the left. ZapA dimers are sparsely bound to the FtsZ single protofilament and are only visible as cloud densities as indicated by light blue arrowheads. The residues A11, E33, and K51-G55 of FtsZ involved in the interaction of ZapA with the FtsZ double protofilament are shown as light green spheres in the ribbon model of single protofilament to indicate the positions of FtsZ interface with ZapA. In the ribbon model on the very right, the model of FtsZ double protofilament-ZapA tetramer taken from Supplementary Fig. 9b is also included as a volume map to indicate the possible interaction of ZapA dimer with the FtsZ single protofilament. The dotted arrow indicates the direction of view of the projection map on the second left.

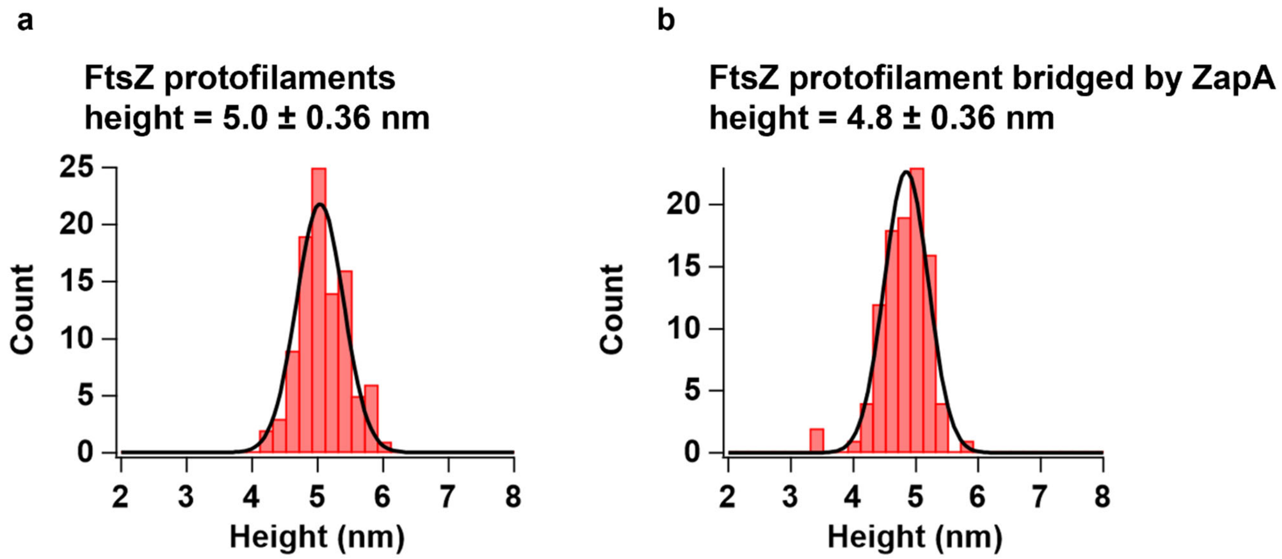

Supplementary Fig. 12: Height histogram of FtsZ protofilaments alone and (**b**) FtsZ filaments cross-bridged by ZapA (n = 100 for both). Solid lines represent Gaussian distributions, with height = center value ± σ. Source data of the graphs are provided as a Source Data file.

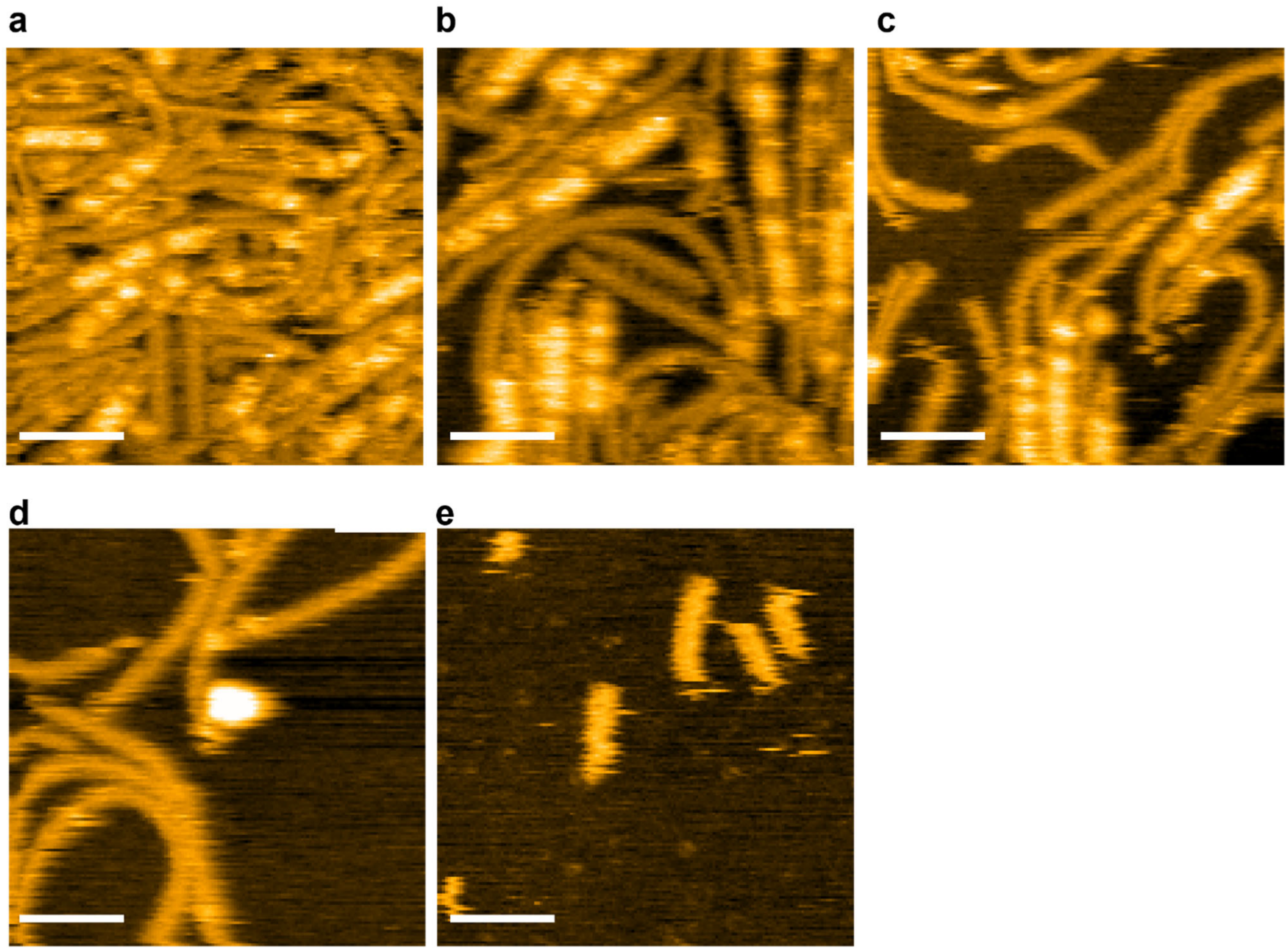

Supplementary Fig. 13: HS-AFM images of FtsZ-ZapA complexes at different concentrations. HS-AFM images after mixing FtsZ (15 μM) and ZapA (30 μM) with (a) no dilution, (b) ×10 dilution, (c) ×20 dilution, (d) ×50 dilution and (e) ×100 dilution. Scale bar: 50 nm, frame rate: 1 frame per second (fps), 200 × 200 pixels. Similar results were obtained from more than 5 independent experiments.

Supplementary Table 1: Cryo-EM data collection and processing

Dataset	FtsZ double filament-ZapA tetramer
EMDB accession code	EMD- 60837
PDB accession code	9ISK
**Data collection and processing**	
Magnification	60,000
Voltage (kV)	300
Electron exposure ($e^{-}/Å^{2}$)	60
Defocus range (μm)	−0.5 to −2.0
Pixel size (Å)	0.878
Symmetry imposed	D1 helical
Imported movies (no.)	7,375
Initial particle images (no.)	4,374,348
Final particle images (no.)	54,670
Map resolution (Å)	2.73
FSC threshold	0.143
Helical parameters	
Rise (Å)	44.58
Twist (°)	-3.11
**Refinement**	
Initial model used (PDB code)	8IBN, 4P1M
Model resolution (Å)	2.6/2.7/3.0
FSC threshold	0/0.143/0.5
Model vs. Data CC (mask)	0.84
(volume)	0.84
Model composition	
Non-hydrogen atoms	17,400
Protein residues	2,324
Ligands	6 (GMPCPP)
	6 (Mg)
	6 (K)
R.m.s. deviations	
Bond lengths (Å)	0.005
Bond angles (°)	0.626
Validation	
MolProbity score	1.76
Clashscore	9.97
Rotamer outliers (%)	0
Ramachandran plot	
Favored (%)	96.43
Allowed (%)	3.57
Outliers (%)	0

Supplementary Table 2: Protein-protein interface analysis by PDBePISA server.

Chain 1:Chain 2	Interface, Å^2	$\Delta^i G$, kcal
ABEF:GH	2105.4	–17.0
A:B	1390.6	–16.0
B:EF	523.4	–3.6
B:F	207.7	1.0
B:E	315.7	–4.6
ABC:DEF	1268.2	–10.6

Supplementary Table 3: Crystallographic data collection and refinement statistics.

Dataset	KpZapA
PDB accession code	9ISJ
**Data collection and processing**	
Space group	$P6_122$
Cell dimensions	
a, *b*, *c* (Å)	54.173, 54.173, 330.044
α, β, γ (°)	90.00, 90.00, 120.00
Total reflections	250,986 (22,250)*
Unique reflections	28,055 (2,712)
Resolution range	46.45–1.80 (1.86–1.80)
R_{merge}	0.05109 (1.101)
$I/\sigma I$	20.03 (1.66)
Completeness (%)	99.84 (100.0)
Redundancy	9.0 (8.2)
$CC_{1/2}$	0.999 (0.872)
**Refinement**	
R_{work}/R_{free}	0.215/0.239
No. of atoms	
Protein	1712
Ligand	1
Water	134
B factors (Å^2)	
Protein	45.74
Ligand	57.39
Water	48.94
R. m. s. deviations	
Bond length (Å)	0.0089
Bond angles (°)	1.6067
Validation	
MolProbity score	0.95
Clashscore	1.75
Rotamer outliers (%)	1.06
Ramachandran plot	
Favored (%)	100.00
Allowed (%)	0.00
Outliers (%)	0.00

*Each dataset was collected from one crystal. *Values in parentheses are for highest-resolution shell.

Supplementary Table 4: Protein-protein interface analysis by PDBePISA server.

KpFtsZ	**$\Delta^{i}G$, kcal mol^{-1}**	**Difference in $\Delta^{i}G$, kcal mol^{-1} from WT**
WT	−17.0	-
F2A	−12.7	4.3
E3A	−17.8	−0.8
P4A	−15.6	1.4
M5A	−13.4	3.6
E6A	−16.0	−2.1
L7A	−17.6	1.0

Supplementary Table 5: List of synthesized genes and primers used in this study and their sequences

Name	Sequence
KpZapA_WT	5′-GAGAATCTTTATTTCCAGGGT<u>CATATG</u>ATGTCTGCTCAACCGGTAGACTTGC<br>AGATCTTTGGCCGTTCACTTCGCGTCAATTGCCCTCCGGAACAACGCGATGCGCT<br>CAATCAGGCGGCTGAAGATCTGAACCAGCGTCTGCAAGACCTGAAAGAGCGTACA<br>CGCGTTACCAACACCGAACAGTTGGTGTTCATCGCAGCACTGAACATCTCGTACG<br>AACTGACTCAGGAGAAAGCCAAAACCCGCGATTATGCCAGTAGCATGGAACAGCG<br>CATTCGGATGTTACAACAGACGATTGAGCAGGCGTTACTGGAACAAGGGCGCATT<br>TCCGAACGTCCAGGTAGCAAGTTTGAATAG<u>TCTAGA</u>TAGGTAATCTCTGCTTAAA<br>AG-3′
pCold-KpZapA_WT_Fw	5′-TCTAGATAGGTAATCTCTGCTTAAAAG-3′
pCold-KpZapA_WT_Rv	5′-CATATGACCCTGGAAATAAAGATTCTC-3′
pCold-KpZapA_I83E_Fw	5′-GAACGGATGTTACAACAGACGATTGAG-3′
pCold-KpZapA_I83E_Rv	5′-GCGCTGTTCCATGCTACTGG-3′
pCold-KpFtsZ_F2A_Fw	5′-GCGGAGCCTATGGAACTCAC-3′
pCold-KpFtsZ_F2A_Rv	5′-CATATGACCCTGGAAATAAAGATTCTCCTG-3′
