# Supplementary material for: Structural basis for the interaction between the bacterial cell division proteins FtsZ and ZapA
Source: Nat Commun. 2025 Jul 1;16:5985. doi: 10.1038/s41467-025-60940-w (PMC12216130; doi:10.1038/s41467-025-60940-w)
Supplement: Supplementary file 2 — Description of Additional Supplementary Files [file 41467_2025_60940_MOESM2_ESM.pdf]

Description of Additional Supplementary Files

Supplementary Movie 1: HS-AFM movie of a mixed sample of FtsZ-WT protofilaments and ZapA. Multiple ZapA molecules are observed bound to the FtsZ protofilaments (ZapA addition method: 0.1 mM GMPCPP, 13 µM KpZapA). Frame rate: 1 fps. ×5 playback.

Supplementary Movie 2: HS-AFM movie of a mixed sample of FtsZ-F2A protofilaments and ZapA (ZapA addition method: 1 mM GMPCPP, 27 µM KpZapA). No ZapA binding is observed on the FtsZ protofilaments. Frame rate: 1 fps. ×5 playback.

Supplementary Movie 3: Wide-field HS-AFM movie showing protofilaments not remaining stationary, but fluctuating on the mica surface due to diffusion. Frame rate: 2 fps. ×5 playback.

Supplementary Movie 4: Wide-field HS-AFM movie of FtsZ-ZapA complex. ZapA addition method: 1 mM GMPCPP, 27 µM KpZapA. Frame rate: 1 fps. ×5 playback.

Supplementary Movie 5: HS-AFM movie demonstrating the dynamic interaction between ZapA and an FtsZ protofilament. Frame rate: 2 fps, 3× playback speed.

Supplementary Movie 6: HS-AFM movie showing the process of ZapA-mediated crosslinking of FtsZ single protofilaments. Zap addition method: 1 mM GMPPNP, 1.3 µM KpZapA. Frame rate: 2 fps. ×3 playback.
