# Supplementary material for: Structural basis for the interaction between the bacterial cell division proteins FtsZ and ZapA
Source: Nat Commun. 2025 Jul 1;16:5985. doi: 10.1038/s41467-025-60940-w (PMC12216130; doi:10.1038/s41467-025-60940-w)
Supplement: Supplementary file 9 — Reporting Summary [file 41467_2025_60940_MOESM9_ESM.pdf]

nature portfolio

Corresponding author(s): Takayuki Uchihashi, Hiroyoshi Matsumura

Last updated by author(s): Jun 4, 2025

Reporting Summary

Nature Portfolio wishes to improve the reproducibility of the work that we publish. This form provides structure for consistency and transparency in reporting. For further information on Nature Portfolio policies, see our Editorial Policies and the Editorial Policy Checklist.

Statistics

For all statistical analyses, confirm that the following items are present in the figure legend, table legend, main text, or Methods section.

n/a	Confirmed	
☐	☒	The exact sample size (n) for each experimental group/condition, given as a discrete number and unit of measurement
☐	☒	A statement on whether measurements were taken from distinct samples or whether the same sample was measured repeatedly
☒	☐	The statistical test(s) used AND whether they are one- or two-sided<br>*Only common tests should be described solely by name; describe more complex techniques in the Methods section.*
☒	☐	A description of all covariates tested
☒	☐	A description of any assumptions or corrections, such as tests of normality and adjustment for multiple comparisons
☐	☒	A full description of the statistical parameters including central tendency (e.g. means) or other basic estimates (e.g. regression coefficient) AND variation (e.g. standard deviation) or associated estimates of uncertainty (e.g. confidence intervals)
☒	☐	For null hypothesis testing, the test statistic (e.g. F, t, r) with confidence intervals, effect sizes, degrees of freedom and P value noted<br>*Give P values as exact values whenever suitable.*
☒	☐	For Bayesian analysis, information on the choice of priors and Markov chain Monte Carlo settings
☒	☐	For hierarchical and complex designs, identification of the appropriate level for tests and full reporting of outcomes
☒	☐	Estimates of effect sizes (e.g. Cohen's d, Pearson's r), indicating how they were calculated

Our web collection on statistics for biologists contains articles on many of the points above.

Software and code

Policy information about availability of computer code

Data collection	All the cryoEM image datasets were collected using SerialEM ver. 4.0, yoneoLocr ver. 1.0, and CRYO ARM 300 or CRYO ARM 300 (JEOL). X-ray diffraction data were collected on the micro-focus beamline BL41XU at SPring-8, Hyogo, Japan using an EIGER X 16M detector (Dectris). HS-AFM observations were performed using a laboratory-built system: https://doi.org/10.1007 /s00424-007-0406-0 .
Data analysis	CryoEM: RELION ver. 4.0, cryoSPARC ver. 3.3.1 and 4.1.1, Coot ver. 0.9.6, PHENIX ver. 1.19.2, MolProbity ver. 4.5.2, UCSF Chimera ver. 1.15, ChimeraX ver. 1.1, PyMOL ver. 2.5.0, LigPlot+ ver. 2.2.4, ImageJ ver. 1.53q. X-ray: KAMO system (which runs BLEND, XDS, and XSCALE ver. 5 (February 2021), MOLREP and REFMAC ver. 5.8.0267 in the CCP4 suite ver. 7.1, PHENIX ver. 1.19.2, Coot ver. 0.8.6, MolProbity ver. 4.5.2.<br>HS-AFM image and movie generation, as well as image analysis, were performed using a customized AFM image viewer and analysis software based on Igor Pro (Version 9.0) provided by WaveMetrics Inc.

For manuscripts utilizing custom algorithms or software that are central to the research but not yet described in published literature, software must be made available to editors and reviewers. We strongly encourage code deposition in a community repository (e.g. GitHub). See the Nature Portfolio guidelines for submitting code & software for further information.

Data

Policy information about availability of data

All manuscripts must include a data availability statement. This statement should provide the following information, where applicable:

- Accession codes, unique identifiers, or web links for publicly available datasets
- A description of any restrictions on data availability
- For clinical datasets or third party data, please ensure that the statement adheres to our policy

Cryo-EM atomic coordinates and maps of K. pneumoniae FtsZ double filament-ZapA tetramer have been deposited in the Protein Data Bank (PDB) and the Electron Microscopy Data Bank (EMDB) under the accession codes 9ISK and EMD-60837. Coordinates and structure factors of K. pneumoniae ZapA have been deposited in PDB under the accession number 9ISJ. The other coordinates used in this study are available from PDB. Source data are provided in this paper.
The data necessary to evaluate the conclusions related to the HS-AFM Data in this study are provided in the main text, including the main figures, as well as in the Supplementary Information. Source data for the graphs obtained from the analysis of HS-AFM images are included in the Source Data file.
Further analytical or interpretive support is available from the corresponding author upon request.

Research involving human participants, their data, or biological material

Policy information about studies with human participants or human data. See also policy information about sex, gender (identity/presentation), and sexual orientation and race, ethnicity and racism.

Reporting on sex and gender	N/A
Reporting on race, ethnicity, or other socially relevant groupings	N/A
Population characteristics	N/A
Recruitment	N/A
Ethics oversight	N/A

Note that full information on the approval of the study protocol must also be provided in the manuscript.

Field-specific reporting

Please select the one below that is the best fit for your research. If you are not sure, read the appropriate sections before making your selection.

[x] Life sciences [ ] Behavioural & social sciences [ ] Ecological, evolutionary & environmental sciences

For a reference copy of the document with all sections, see nature.com/documents/nr-reporting-summary-flat.pdf

Life sciences study design

All studies must disclose on these points even when the disclosure is negative.

Sample size	In the cryoEM study, totally 4,374,348 particle images were picked from 6,070 micrographs for the ZapA-FtsZ complex. 94,670 particle images were used for final reconstruction, because they were classified into higher resolution subsets. Similarly, totally 3,409,185 particle images were picked from 5,285 micrographs for the ZapA (I83E)-FtsZ complex. A total of 628,514 particle images were used among the 2D class averages of different orientations. These were also classified into higher-resolution subsets. For other experiments, the sample sizes were chosen based on the reproducibility and variation of the data.<br>Sample sizes were not predetermined in this study, as the analysis was based on high-speed AFM images we obtained. Conclusions were reached using all available data of sufficient quality.
Data exclusions	In the cryoEM study of the ZapA-FtsZ and ZapA (I83E)-FtsZ complex, 4,319,678 and 2,780,671 particle images were excluded from the initial 4,374,348 and 3,409,185 particle images, respectively.<br>Since the quality of high-speed AFM images depends on the AFM tip condition and other experimental factors, we manually excluded particles showing poor resolution or high noise level to ensure detailed observation.
Replication	For the negative staining, each experiment was repeated independently at least twice with similar results. For the cryoEM study, two grids were prepared to obtain similar results. Each structural analysis of cryoEM and X-ray was performed from the single dataset. For the SDS-PAGE analysis, the experiment was repeated three times with similar results. For HS-AFM, each experiment was repeated independently at least 5 times with similar results.
Randomization	Randomization is not relevant to structural analysis and HS-AFM analysis, as the protein is not required to be allocated into experimental groups.
Blinding	Blinding is not relevant to this study, as the protein is not required to be allocated into experimental groups in structural analysis.

Reporting for specific materials, systems and methods

We require information from authors about some types of materials, experimental systems and methods used in many studies. Here, indicate whether each material, system or method listed is relevant to your study. If you are not sure if a list item applies to your research, read the appropriate section before selecting a response.

Materials & experimental systems

n/a	Involved in the study
☒	☐ Antibodies
☒	☐ Eukaryotic cell lines
☒	☐ Palaeontology and archaeology
☒	☐ Animals and other organisms
☒	☐ Clinical data
☒	☐ Dual use research of concern
☒	☐ Plants

Methods

n/a	Involved in the study
☒	☐ ChIP-seq
☒	☐ Flow cytometry
☒	☐ MRI-based neuroimaging

Plants

Seed stocks	N/A
Novel plant genotypes	N/A
Authentication	N/A
